# ICAM-2 regulates vascular permeability and N-cadherin localization through ezrin-radixin-moesin (ERM) proteins and Rac-1 signalling

**DOI:** 10.1186/1478-811X-12-12

**Published:** 2014-03-04

**Authors:** Valerie Amsellem, Nicola H Dryden, Roberta Martinelli, Felicity Gavins, Lourdes Osuna Almagro, Graeme M Birdsey, Dorian O Haskard, Justin C Mason, Patric Turowski, Anna M Randi

**Affiliations:** 1 Imperial College for Translational and Experimental Medicine, NHLI Vascular Sciences, Imperial College London, Hammersmith Hospital, Du Cane Road, London W12, ONN, UK; 2 Cell Biology, UCL Institute of Ophthalmology, 11-43 Bath Street, London EC1V 9EL, UK; 3 Division of Brain Sciences, Burlington Danes building, Imperial College London, Hammersmith Hospital, London, UK; 4 Present address: Institut National de la Santé et de la Recherche Médicale (INSERM) U955 and Département de Physiologie-Explorations Fonctionnelles, Hôpital Henri Mondor, Assistance Publique-Hôpitaux de Paris (AP-HP) and Université Paris Est, Créteil, France; 5 Present address: Cancer Research UK, London, UK; 6 Present address: Center for Vascular Biology Research, Beth Israel Deaconess Medical Center, Harvard Medical School, Boston, MA, USA

**Keywords:** Endothelial, ICAM-2, N-Cadherin, ERM, Rac-1, Cell adhesion, Permeability, Cell-cell junctions

## Abstract

**Background:**

Endothelial junctions control functions such as permeability, angiogenesis and contact inhibition. VE-Cadherin (VECad) is essential for the maintenance of intercellular contacts. In confluent endothelial monolayers, N-Cadherin (NCad) is mostly expressed on the apical and basal membrane, but in the absence of VECad it localizes at junctions. Both cadherins are required for vascular development. The intercellular adhesion molecule (ICAM)-2, also localized at endothelial junctions, is involved in leukocyte recruitment and angiogenesis.

**Results:**

In human umbilical vein endothelial cells (HUVEC), both VECad and NCad were found at nascent cell contacts of sub-confluent monolayers, but only VECad localized at the mature junctions of confluent monolayers. Inhibition of ICAM-2 expression by siRNA caused the appearance of small gaps at the junctions and a decrease in NCad junctional staining in sub-confluent monolayers. Endothelioma lines derived from WT or ICAM-2-deficient mice (IC2neg) lacked VECad and failed to form junctions, with loss of contact inhibition. Re-expression of full-length ICAM-2 (IC2 FL) in IC2neg cells restored contact inhibition through recruitment of NCad at the junctions. Mutant ICAM-2 lacking the binding site for ERM proteins (IC2 ΔERM) or the cytoplasmic tail (IC2 ΔTAIL) failed to restore junctions. ICAM-2-dependent Rac-1 activation was also decreased in these mutant cell lines. Barrier function, measured i*n vitro* via transendothelial electrical resistance, was decreased in IC2neg cells, both in resting conditions and after thrombin stimulation. This was dependent on ICAM-2 signalling to the small GTPase Rac-1, since transendothelial electrical resistance of IC2neg cells was restored by constitutively active Rac-1*. In vivo*, thrombin-induced extravasation of FITC-labeled albumin measured by intravital fluorescence microscopy in the mouse cremaster muscle showed that permeability was increased in ICAM-2-deficient mice compared to controls.

**Conclusions:**

These results indicate that ICAM-2 regulates endothelial barrier function and permeability through a pathway involving N-Cadherin, ERMs and Rac-1.

## Lay abstract

Blood vessels are lined by a single layer of cells, called endothelial cells, which control many critical functions in the body. Endothelial cells form a tight layer thanks to the presence of proteins on the surface of the cells, which bind to similar proteins on neighboring cells. These proteins, called adhesion molecules, control the flux of liquid from inside the vessels to the tissue. This process, called permeability, is essential for the health of tissues and for the ability of the body to respond to changes in blood pressure or to inflammation. Control of this process can be disturbed in diseases such as cancer or chronic inflammation. Here we identify a new mechanism that controls permeability, which is mediated by a protein called ICAM-2. This protein is also important in regulating inflammation and the formation of new blood vessels, called angiogenesis. We show that ICAM-2 regulates the localization of another adhesion molecule, N-Cadherin, at the endothelial points of cell-cell contact (endothelial junctions). This occurs through signals inside the cells mediated by ICAM-2. These results could have implications for the regulation of vascular permeability in particular areas of the body, such as the eye, and in cancer.

## Introduction

Endothelial intercellular junctions play an essential role in many critical processes, including survival, angiogenesis and inflammation [1-3]. A large number of cell adhesion molecules are localized at the cell junctions; their extracellular domains support intercellular adhesion through homophilic and/or heterophilic interactions, whilst connection to the actin cytoskeleton contributes to strengthening the intercellular contacts. Complex signaling networks regulated by cell-cell junctions control contact inhibition, cell growth, survival and permeability [2,4]. These pathways are modulated by cross-talk between different junctional adhesion molecules and with other surface receptors.

Cadherins are a large family of Ca^2+^-dependent adhesion molecules, which mediate homophilic adhesion through formation of multimeric complexes at the adherens junctions (AJ) [5] and are essential for the maintenance of intercellular contacts. The two main endothelial cadherins, VE-Cadherin (VECad) and N-Cadherin (NCad), share similar structures and intracellular binding partners; however they display differences in tissue distribution and function. VECad expression is restricted mainly to endothelial cells (EC), whilst NCad is expressed in several cell types including neurons, muscle cells and fibroblasts [6-9]. Both cadherins are involved in vascular development and angiogenesis: endothelial deletion of either VECad or NCad leads to embryonic lethality due to vascular defects [10,11].Whilst the role of VECad in controlling endothelial junction stability and function has been intensely investigated, the role of NCad is less clear. In mature stable vessels, VECad is concentrated at endothelial cell-cell junctions and is essential for junction stability, whilst NCad is mainly localized across the cell surface. VECad has been shown to exclude NCad from mature junctions, through pathways that involve β-catenin and p120^ctn^[12,13] and to negatively regulate NCad levels [13]. Despite this, some NCad has been reported at endothelial junctions and shown to be upstream of VECad during blood vessel morphogenesis [14]. Moreover, in specialized endothelial cells such as corneal endothelium, NCad is clearly mainly localized at cell-cell junctions and controls permeability [15].

ICAM-2, a transmembrane glycoprotein of the immunoglobulin superfamily expressed by endothelial cells, platelets and leukocytes [16], is found on the apical surface of endothelial cells and at cell-cell junctions. Like many other junctional adhesion proteins, ICAM-2 can support homophilic adhesion [17,18]. As well as regulating leukocyte adhesion and transmigration [17,19,20], ICAM-2 is required for angiogenesis [17]. In addition, ICAM-2 regulates activation of the small GTPase Rac1 in EC [17]. The short intracellular domain of ICAM-2 is linked to the cytoskeleton through interaction with α-actinin [21] and with members of the ERM family [22]. The ERM proteins are implicated in the regulation of cell-cell and cell-matrix adhesion, partly through the small GTPases pathway (for review [23]).

In this study we investigate the role of ICAM-2 in controlling endothelial junctions and barrier function. We show that ICAM-2 regulates early junction stability and the transient accumulation of NCad at endothelial junctions in loosely established contacts. We also show that ICAM-2 regulates NCad localization through intracellular pathways involving the ERM binding motif and Rac-1 signalling. Finally, we demonstrate that ICAM-2 controls barrier function and vascular permeability *in vitro* and *in vivo*. Thus we identify a novel connection in the complex network regulating localization and signaling at endothelial junctions.

## Results

### Localization of ICAM-2 and NCad on human EC in sub-confluent vs confluent monolayers

**Figure 1:**
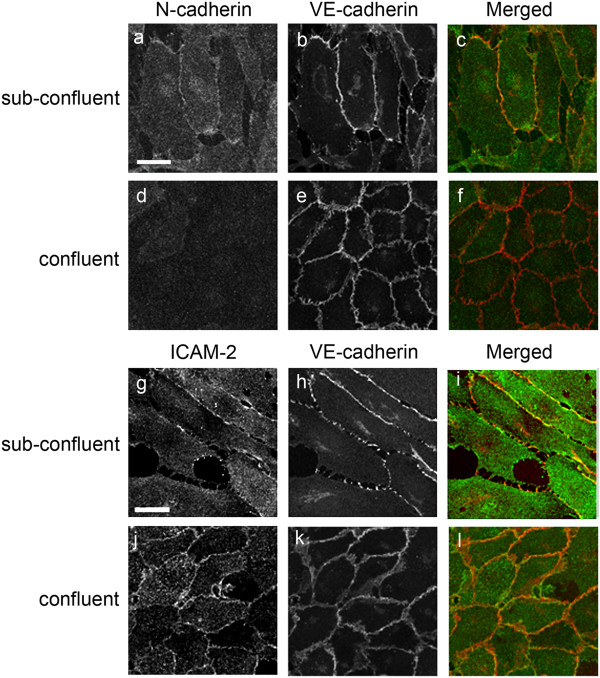
**Distribution of ICAM-2, VECad and NCad in sub-confluent vs confluent HUVEC monolayers.** Cells were seeded at low confluence (10000 cells/cm2) and cultured for 24 and 96 hours to achieve sub confluent and confluent endothelial monolayer respectively. For NCad/VECad co-staining **(a-f)**, NCad was stained using mAb Cl32 anti-NCad followed by anti-mouse AlexaFluor488 (Green) and VECad was stained using mAb Cl55-7H1 anti-human VECad prelabelled with the Zenon® mouse IgG1 555 kit (Red). For ICAM-2/VECad co-staining **(g-l)**, ICAM-2 was visualized using mAb BT-1 followed by anti-mouse AlexaFluor 488 (Green) and VECad was stained as described above. Co-staining for ICAM-2 and NCad was attempted with several antibodies and methods, however due to species incompatibility and the well known limitations of NCad antibodies available, satisfactory images could not be achieved. Bar = 25 μm.

The organization of AJ can be different at different stages of confluence and maturity; therefore we set out to investigate the localization of ICAM-2 and the two cadherins on EC by performing co-staining in HUVEC monolayers at different stages of confluency. In monolayers just beginning to reach confluency (referred to as “sub-confluent” from now on), both VECad and NCad showed discontinuous staining at cell-cell contacts (Figure 1, panel a, b and h); NCad was also distributed over the apical and basal surface of the cells (Figure 1, panel a). In confluent monolayers, VECad accumulated at the junctions to form the characteristic thick zipper-like structure (Figure 1, panel e and k) whilst NCad was no longer at cell-cell contacts (Figure 1, panel d) and its expression levels were downregulated (Additional file 1: Figure S1). ICAM-2 was localized at cell-cell contacts and on the apical surface both in sub-confluent and confluent monolayers (Figure 1, panel g and j). These data indicate that in sub-confluent HUVEC monolayers both VECad and NCad can be found at the junctions, and suggest that the localization and expression of N-cadherin in endothelial cells is partly due to the maturation stage of adherens junctions. ICAM-2 and VECad, on the other hand, appear to be localized at cell junctions in both sub-confluent and confluent monolayers, with staining intensity increasing over time.

### Distribution of NCad in early endothelial cell-cell junctions is regulated by ICAM-2

**Figure 2:**
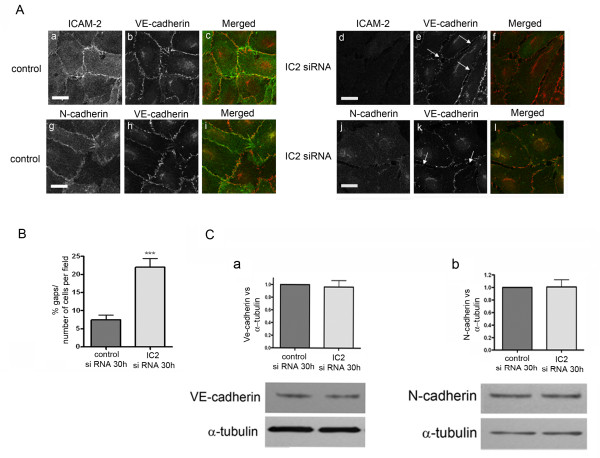
**Distribution of NCad in early endothelial cell-cell junctions is regulated by ICAM-2. A**- Distribution of ICAM-2, VECad and NCad in HUVEC ~30 hours post siRNA treatment (**a-i**: control siRNA; **d-l**: ICAM-2 siRNA). Arrows indicate the gaps between cells that transiently appeared following ICAM-2 inhibition by siRNA. For ICAM-2/VECad co-staining (**a-f**), see details in Figure 1 legend. Bar = 25 μm **B**- Quantification of the number of gaps. The gaps were counted manually from 15 fields taken from confocal immunofluorescence images (233 μm x 233 μm, see panel **B)** in two independent experiments. Results are shown as % of gaps per number of cells per field. Error bars indicate mean ± S.E.M., n = 30. Statistical analysis (t-test: ***p < 0.001). **C**- VECad and NCad levels are unchanged 30 h post-treatment with ICAM2 siRNA. Quantification of VECad and NCad: Western blot quantification was performed by densitometry, normalized to α-tubulin. Error bars indicate mean ± s.e.m., n = 5.

To test whether ICAM-2 may be involved in regulating endothelial junction assembly and/or integrity, we inhibited ICAM-2 expression in HUVEC using ICAM-2 siRNA. This resulted in a significant decrease in ICAM-2 protein levels (Additional file 2: Figure S2A) and cell surface staining (Figure 2A, panel d). Morphological analysis showed that in loosely confluent areas inhibition of ICAM-2 expression (30 h after siRNA treatment) disrupted the continuity of the early established cell-cell contacts, with the appearance of gaps (Figure 2A, panel d-l). Quantification of these areas showed that the number of junctional gaps in ICAM-2 siRNA-treated cells was significantly higher compared to cells treated with control siRNA (Figure 2B). VECad levels were not affected by the loss of ICAM-2 (Figure 2C); VECad junctional staining appeared more fragmented but not significantly altered by ICAM-2 siRNA treatment (Figure 2A, panels e and k). On the other hand, in ICAM-2 deficient cells, NCad localization at the cell junctions of loosely confluent cells was lost (Figure 2A, panel j) without modification of NCad level (Figure 2C). Loss of junctional localization was not a generalized phenomenon, since staining for CD31/PECAM was similar in ICAM-2 and control siRNA-treated cells (Additional file 3: Figure S3). These data indicate that ICAM-2 is involved in regulating NCad localization in recently established endothelial junctions and is required for junction assembly in primary EC.

### ICAM-2 regulates N-Cad localization at cell-cell junction

**Figure 3:**
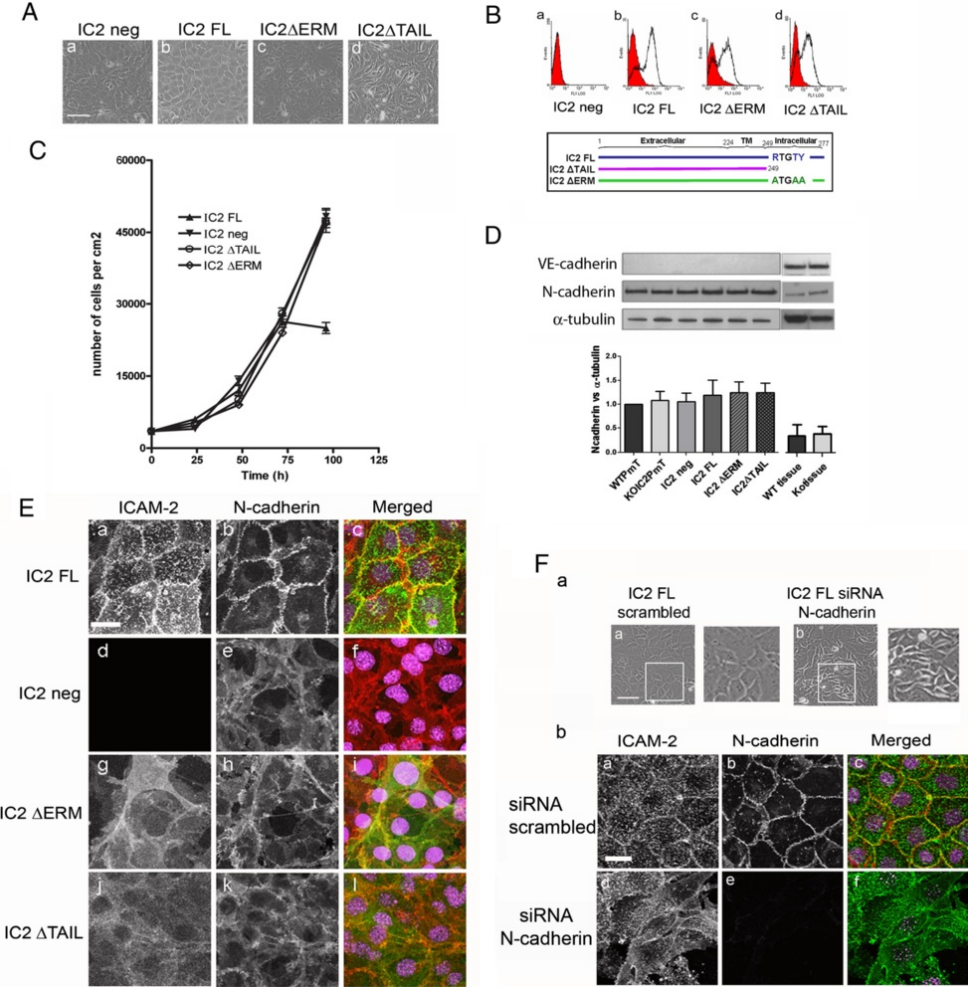
**ICAM-2 regulate N-Cad localization at cell-cell junction. A**- Generation of mouse cardiac EC (MCEC) cell lines. Phase contrast image of IC2 neg **(a)**, IC2FL **(b)**, IC2 ΔERM **(c)** and IC2 ΔTAIL **(d)** cells. Bar = 150 μm. **B**- Analysis of ICAM-2 surface protein expression by FACS in IC2 neg **(a)**, IC2 FL **(b)**, IC2 ΔERM **(c)**, IC2 ΔTAIL **(d)** cells; below, diagram showing the WT and mutant sequences of the constructs. FACS staining was performed using mAb 3C4 anti-mouse ICAM-2. **C**- Growth curves of MCEC cell lines. IC2 neg, IC2 ΔERM and IC2 ΔTAIL cells show loss of contact inhibition of cell growth. Results are represented as number of cells/cm^2^ over time (hours). **D**- Western blot analysis of NCad and VECad levels in IC2 neg, IC2 FL, IC2 ΔERM and IC2 ΔTAIL endothelioma lines and heart tissue from WT and IC2 deficient mice (ko). VECad and NCad were detected using mAb BV13 and mAb Cl32, respectively. Error bars indicate mean ± s.e.m., n = 3. **E**- Analysis of ICAM-2 and NCad distribution in IC2 FL **(a-c)**, IC2 neg **(d-f)**, IC2 ΔERM **(g-i)** and IC2 ΔTAIL **(j-l)** cells. ICAM-2 was stained with mAb 3C4 anti-mouse ICAM-2 followed by anti-rat AlexaFluor488 (Green). NCad was stained with mAb Cl32 anti-NCad followed by anti-mouse AlexaFluor555 (Red). Nuclei were stained using TOPRO-3 (Purple). Bar = 25 μm. **F**- Inhibition of NCad expression in IC2 FL cell line by siRNA resulted in disruption of cell-cell contacts and altered cell morphology. **(a)**- Phase contrast image of scrambled siRNA **(a)** and NCad siRNA **(b)** 48 hours post-transfection, Bar = 150 μm. **(b)**- Localization of ICAM-2 and NCad in IC2 FL cells treated with 48 hours scrambled **(a-c)** or NCad siRNA **(d-f)**.

To investigate whether ICAM-2-dependent signaling is involved in regulating NCad localization in EC, we generated ICAM-2 endothelioma lines from WT and ICAM-2-deficient mice. EC isolated from cardiac tissue by positive selection [17] were immortalized by infection with the polyoma virus middle-T oncogene [24]. The endothelial lineage of puromycin selected lines was confirmed by endoglin surface expression (Additional file 4: Figure S4C) and by functional analysis of tube formation in matrigel (Additional file 4: Figure S4B). Immortalization of mouse EC with this approach resulted in loss of VECad expression (Figure 3D); this allowed us to use these lines to investigate the function of NCad at the endothelial junctions. Interestingly, PECAM-1 expression was also lost in these lines, whilst expression of JAM-A was retained (data not shown).

The ICAM-2-deficient endothelial line (KOIC2-PmT) was used for re-expression of murine wild type full length (IC2 FL) or mutant ICAM-2. After infection of pBabe-IC2, IC2 FL cells expressed ICAM-2 surface levels similar to those found in HUVEC (Additional file 4: Figure S4C). In these cells ICAM-2 was strongly localized at cell-cell junctions; intense ICAM-2 staining on the apical surface of the cells was also observed (Figure 3E, panel a), as expected [25]. Previous work has shown that ICAM-2 deficiency in EC result in reduced tube formation in Matrigel [17]. Tube network formation was restored in IC2 FL cells, compared to IC2 neg cells (Additional file 4: Figure S4B), indicating that the phenotype of the IC2 neg cells can be reversed by re-expression of ICAM-2.

A striking difference in cell morphology between IC2 neg and IC2 FL mouse cell lines was observed. IC2 neg cells were unable to form a confluent cobblestone monolayer; organized junctions between cells were lost and the cells grew on top of each other, suggesting loss of contact inhibition (Figure 3A, panel a). Cobblestone morphology and junction formation were restored by over-expression of ICAM-2 in the IC2 FL cells (Figure 3A panel b). To test whether the disruption of the monolayer resulted in disruption of cell growth, we measured the proliferation rate of the two endothelial lines. As shown in Figure 3C, proliferation of IC2 FL cells reached a plateau at 72 hours, whilst IC2neg cells continued to proliferate. These findings indicate loss of contact-dependent inhibition of cell growth in IC2 neg cells. In these cells, VECad disappears after immortalization ([26] and Figure 3D), whilst NCad is expressed at high levels. NCad protein levels were similar in both lines (Figure 3D). NCad staining was diffusely expressed on the cell surface of IC2 neg cell line (Figure 3E, panel e); however, when ICAM-2 expression was restored (IC2 FL), NCad was recruited to the junctions (Figure 3E, panel b).

To test whether NCad was responsible for junction assembly and contact inhibition of cell growth in these cells, NCad expression was inhibited in the IC2 FL line using siRNA (Figure 3F and S2B). Inhibition of NCad expression in IC2 FL cells resulted in loss of normal cobblestone appearance (Figure 3F, panel b). The cells showed irregular morphology and loss of contact inhibition; ICAM-2 staining was distributed on the apical surface and in some areas around the edge of the cells (Figure 3F, panel b). Thus, in the absence of VECad, NCad localizes at cell-cell junctions, where it regulated contact inhibition and junction assembly. ICAM-2 is required for NCad localization at the junctions; deficiency of ICAM-2 results in loss of NCad junctional localization and, in the absence of VECad, loss of contact inhibition and junction assembly.

### ERMs are involved in the ICAM-2-dependent N-cadherin recruitment and junction formation

ICAM-2 regulates several signal transduction pathways. Ligation of ICAM-2 in leukocyte and fibroblast lines resulted in activation of the PI-3K/Akt pathway and inhibition of apoptosis, through involvement of ERM proteins [27]. To test whether ICAM-2 intracellular signaling and interaction with ERM proteins are involved in recruitment of NCad to the junctions, we generated two ICAM-2 mutants, one lacking the 28 amino acids (aa 249-277) corresponding to the cytoplasmic tail (IC2 ΔTAIL), and one where the ERM binding site (residues 255 to 264, RxxTYxVxxA) was mutated (IC2 ΔERM). To abolish ERM binding without affecting the α-actinin binding site, only the first three key residues R, T and Y of the ERM binding motif were mutated to alanines (Figure 3B). Individual loss of these residues has previously been shown to abrogate interaction between ICAM-2 and ERMs [28]. The mutant ICAM-2 cDNAs were cloned into pBabe retroviral vectors and used to infect the IC2 neg-PmT endothelioma line (see above). Surface expression levels of ICAM-2 in the IC2 FL, IC2 ΔERM and IC2 ΔTAIL lines were similar (Figure 3B). When examined by phase contrast microscopy (Figure 3A), IC2 ΔERM and IC2 ΔTAIL cells were unable to form cobblestone monolayers, similar to IC2 neg cells. Both IC2 ΔERM and IC2 ΔTAIL cells showed an irregular and elongated morphology with projections often protruding over neighboring cells; this was more pronounced in the IC2 ΔTAIL mutant line (Figure 3A and 3E, panel g and j). Proliferation curves of both mutant cell lines resembled that of the IC2 neg cells, with exponential growth and no plateau (Figure 3C), indicating loss of contact inhibition. We next investigated the cellular localization of ICAM-2 and NCad in the cell lines. In IC2 ΔERM and IC2 ΔTAIL cells, ICAM-2 was diffusely expressed over the apical surface and on the irregular cytoplasmic projections (Figure 3E). In both mutant lines, NCad was also diffusely expressed on the cell surface, similar to the pattern observed in the IC2 neg line (Figure 3E). Co-localization of NCad and ICAM-2 is clearly visible in areas of cell-cell contact. Expression levels of NCad were comparable across all IC2 lines (Figure 3D). These results indicate that ICAM-2 localization at the junctions is dependent on its cytoplasmic tail and on the interaction with ERM proteins. Moreover, NCad localization and junction assembly in these cells requires intracellular signaling through ICAM-2 and ERMs.

### ICAM-2 interaction with ERM proteins regulates Rac1 activity

**Figure 4:**
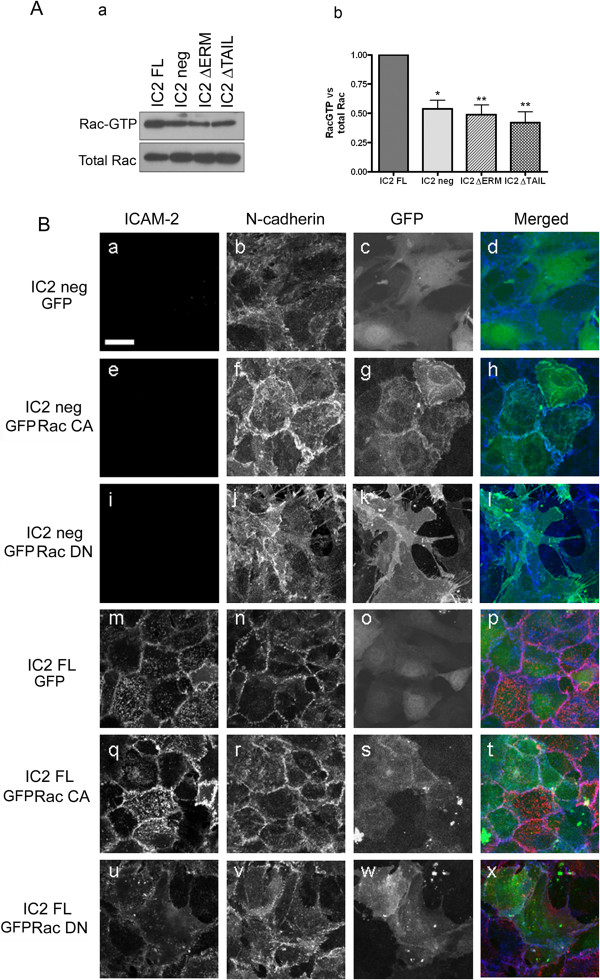
**ERMs and Rac1 activity are involved in ICAM-2-dependent NCad recruitment and cell-cell contact. A**- Rac1 activity in MCEC cell lines, as measured by the GST-PAK pull-down assay. **(a)** Representative Western blot analysis of Rac1 activity. Rac1-GTP pull-down with GST-PAK as well as total Rac1 were detected using mAb Cl23A8. **(b)** Quantification of Rac1 activity (Rac1-GTP/Total Rac1) in IC2 neg, IC2 FL, IC2 ΔERM, IC2 ΔTAIL cell lines. Error bars indicate mean ± s.e.m., n = 6. Statistical analysis (t-test: *p < 0.05, **p < 0.005). **B**- Effect of DN and CA Rac1 on the morphology and the distribution of NCad in the IC2 neg and IC2 FL lines. IC2 neg or IC2 FL were transfected with pEGFP control (**a-d** and **m-p**), pEGFP-V12 Rac1 (**e-h** and **q-t**) or pEGFP-V12-N17 Rac1 (**i-l** and **u-x**). Cells were co-stained for ICAM-2 and NCad. ICAM-2 was stained using the mAb 3C4 anti-mouse ICAM-2 followed by anti-rat AlexaFluor488 (pseudo colored Red). NCad was stained using mAb Cl32 anti-NCad followed by anti-mouse AlexaFluor555 (pseudo colored Blue). Bar = 25 μm.

Rac1 is key regulator of the actin cytoskeleton, cell motility, junction assembly and stability and can modulate assembly and disassembly of adherens junctions [28-32]. We have previously shown that ICAM-2 cross-linking in EC results in increased activation of the small GTPase Rac1, and that Rac1 activity is decreased in ICAM-2 deficient cells [17]. As expected, IC2 neg cells showed decreased levels of Rac1 activity compared to IC2 FL cells (Figure 4A). To determine whether the activation of Rac1 by ICAM-2 is dependent on ICAM-2 binding to ERMs, we measured Rac1 activity in the IC2 ΔERM and IC2 ΔTAIL cell lines. In both cell lines Rac1 activity was decreased compared to IC2 FL (Figure 4A). These data indicates that ICAM-2 mediates activation of Rac1 in EC via a signaling pathway involving ERMs.

### Rac1 is involved in ICAM-2-dependent recruitment of NCad and junction assembly

Rac1 activity is required to assemble adherens junctions, in a complex and dynamic pathway. To establish whether Rac1 is involved in the ICAM-2 and NCad-dependent regulation of endothelial junction stability, we made use of two well characterized mutants of Rac1, V12-N17 and V12, which are respectively a dominant negative (DN) and a constitutively active form (CA) of Rac1 [33]. IC2 neg and IC2 FL cells were transfected with plasmids expressing DN Rac1-GFP or CA Rac1-GFP; empty GFP vector or mock transfections were used as controls. Similar expression levels were obtained in each cell type (data not shown). In IC2 neg cells, over-expression of CA Rac1-GFP, but not DN Rac1-GFP or GFP alone, resulted in restoration of junctions and cobblestone morphology (Figure 4 B, panels a-l). CA Rac1-GFP was concentrated at the cell-cell contacts, as expected [34] (Figure 4 B, panel g). In contrast, disruption of Rac1 activity in IC2 FL cells by DN Rac1-GFP resulted in loss of the regular cobblestone appearance and of cell-cell contacts (Figure 4 B, panels m-x). Over expression of CA Rac1 or GFP alone in IC2 FL cells did not affect the cobblestone appearance (Figure 4B, panels s and o, respectively). In line with these findings, NCad junctional staining, normally absent in IC2 neg cells (see Figure 3E), was restored by over-expression of CA Rac1-GFP (Figure 4B, panel f), but not by DN Rac1-GFP (Figure 4B, panel j). These results indicate that ICAM-2 activation of Rac1 is involved in NCad localization and endothelial junction formation.

### ICAM-2 is controls barrier function and vascular permeability

**Figure 5:**
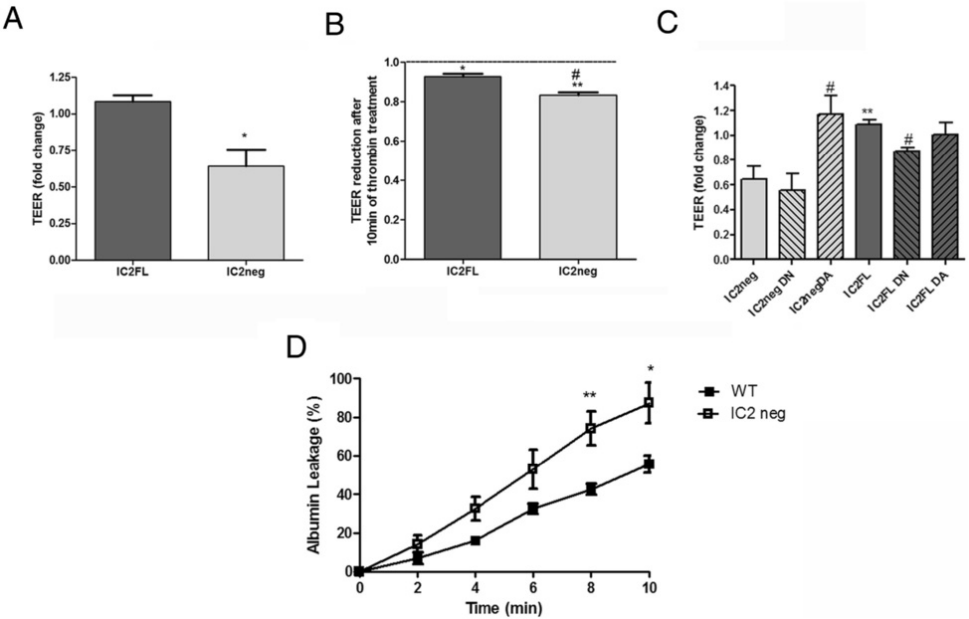
***In vitro *and *in vivo *cell permeability assays. A**- Cells were grown in full medium on gold electrodes until stable impedance was reached. TEER changes were recorded after 48 h and changes in TEER at baseline or **B**- after 10 min thrombin stimulation. Shown are average values -/+ s.e.m. of 4 independent experiments. **C**- Cells were grown in full medium on gold electrodes until stable impedance was reached. Cells were then infected with recombinant adenovirus encoding constitutively active (DA) or dominant negative (DN) myc-Rac1 at an m.o.i. of 400. After 4 h the virus was removed and cells starved to assess barrier development. TEER changes were recorded after 48 h. Shown are average values -/+ s.e.m of 4 independent experiments. **D**- In vivo permeability assay: albumin leakage in postcapillary venules from the cremaster muscles of WT and ICAM-2 KO (IC2 neg) mice in response to thrombin (2U/mL) during 10 min. Data are mean ± s.e.m. of n = 6 mice per group. t-test WT vs IC2-/- *p < 0.05, **p < 0.01.

The results shown so far indicate a role for ICAM-2 in regulating endothelial junction stability and the recruitment of NCad. To test whether ICAM-2 controls endothelial barrier function, we measured transendothelial electrical resistance (TEER) by impedance spectroscopy in IC2 neg and IC2 FL lines. Baseline TEER was significantly higher in IC2FL cells compared to IC2 neg cells (Figure 5A). Thrombin treatment induced a reduction of TEER in all endothelioma cell lines but barrier breakdown was significantly stronger in IC2 neg than IC2 FL cells (Figure 5B) indicating that the baseline endothelial barrier was weaker and also that the barrier was more susceptible to permeabilising agents in the absence of ICAM-2. Rac1 has been shown to control endothelial permeability [35,36]. To determine whether Rac1 was involved in ICAM-2-dependent barrier regulation, we analyse the transendothelial electrical resistance in IC2 neg and IC2 FL cells, infected with recombinant adenovirus expressing constitutively active (DA) or dominant negative Rac1 (DN). In IC2neg cells, DA Rac1 restored the transendothelial electrical resistance to a level similar to IC2 FL, whilst DN had no effect. On the other hand, DN Rac significantly decreased the transendothelial electrical resistance in IC2 FL, whilst DA had no effect (Figure 5C).

Finally, we tested the role of IC2 in regulating vascular permeability *in vivo*, by measuring the extravasation of FITC-labeled albumin via intravital fluorescence microscopy in postcapillary venules of the mouse cremaster muscle. Thrombin caused an increase in FITC-labeled albumin into the adjacent tissue. This effect was significantly enhanced in mice lacking IC2 vs. WT counterparts (Figure 5D) suggesting that the absence of ICAM-2 increases vascular permeability. Taken together these data indicated that the absence of ICAM-2 *in vitro* or *in vivo* increases vascular permeability.

## Discussion

In this study, we present new evidence that the adhesion molecule ICAM-2 is involved in junction stability and the control of permeability by recruiting NCad to the junctions, through pathways which involve ERM proteins and the small GTPase Rac1.

Staining for ICAM-2, NCad and VECad in sub-confluent and confluent HUVEC suggests that NCad junctional localization is transient and occurs at the early stages of cell-cell contact. VECad has been shown to displace NCad from the junctions [12,37,38] and NCad levels are downregulated at confluence [39]. Inhibition of ICAM-2 expression in HUVEC by siRNA resulted in a transient loss of cell-cell contacts and displacement of NCad from the junctions. The transient nature of the disruption of cell junctions caused by ICAM-2 siRNA is likely due to the recruitment and engagement of VECad at the junctions, which over-rides NCad in maintaining junction stability and is seemingly independent of ICAM-2. Therefore we made use of endothelioma mouse lines where VECad expression was permanently lost, to study the role of NCad at the junctions and the role of ICAM2 in regulating its function. The absence of VECad expression from mouse endothelioma lines has not been reported consistently. Loss of VECad expression in endothelioma lines has been observed before [26]; however, endothelioma lines from WT, ICAM-2 or ICAM-1/ICAM-2 double deficient mice were found to express VE-Cad [40,41]. The reason for these discrepancies is unclear. It is conceivable that different protocols for immortalization may be responsible for these differences. Alternatively, or perhaps in combination, the tissue of origin of the cells might influence the ability of the endothelioma lines to retain certain expression profiles. However, in our hands lines from both heart and lung lost VECad expression after passaging. Moreover, three different preparations of endothelioma lines were established and investigated, and all showed the same adhesion molecules’ profile (data not shown).

In non-endothelial tissues, NCad is concentrated at cell-cell contacts where it plays an important role in maintaining barrier function; however the role of NCad at endothelial cell-cell contacts is poorly understood. Several reports show NCad expression in confluent EC monolayers to be diffusely distributed over the surface rather than junctional [37,42]. However, in line with our findings, others have identified NCad expression at endothelial cell-cell junctions and have suggested an indirect role for NCad in regulating junction assembly and stability [14], possibly through the control of VECad expression. The data presented here suggests that NCad may also play a direct, VECad-independent role in maintaining the integrity of immature junctions.

Our data suggest that NCad may be present at immature AJ, possibly during vascular remodeling and/or angiogenesis, or inflammation. AJ organization is different at different stages of cell confluency [43]. Thus, our findings may have implications for neo-vascularization. NCad expression has been associated with neo-vessels in the context of dental inflammation, where the generation of new vessels, in response to dental pulp inflammation, is accompanied by re-expression of NCad in endothelial cells [44]. In tumor angiogenesis, the frequency of hypervascular tumours was shown to be significantly higher for NCad-positive carcinomas than for NCad-negative carcinomas [45]. A direct role for NCad in angiogenesis has been show by Derycke et al, who demonstrated that soluble NCad promotes angiogenesis in both in vitro and in vivo models [46].

Our findings may also be particularly relevant for specialized types of endothelium, such as corneal endothelium (CE). In CE, NCad is a differentiation marker since its expression coincides with the formation of the endothelial cell layer during eye development [15]. Also, in CE the anti-inflammatory neuropeptide VIP up-regulates NCad expression [47]. In the eye, NCad has been shown to regulate vascular permeability [15]. NCad deficiency resulted in disorganization of apical junctional complex in CE, and fluorescein dye injection in the anterior chamber demonstrated increased permeability of the endothelium and corneal edema. To our knowledge, the expression and function of ICAM-2 in CE has not yet been investigated.

In this paper we show that ICAM-2 regulates localization of NCad at the junctions via a pathway involving ERM proteins and the small GTPase Rac1. Over-expression of constitutively active Rac1 restored cell-cell contacts and NCad junctional localization in ICAM-2 deficient cells, and conversely inhibition of Rac1 activity in ICAM-2 over-expressing cells results in monolayer disruption, suggesting that ICAM-2 mediates junction assembly, at least in part, via the Rac1 pathway. Several junctional molecules, including cadherins, have been implicated in Rac1 activation [29,48,49]. The interplay between different stimuli could be critical for time-dependent regulation of Rac1 activation and remains to be investigated. Our data shows that interaction with ERM proteins is required for the ICAM-2-dependent recruitment of NCad at the junctions and contact inhibition of cell proliferation, as well as ICAM-2-dependent activation of Rac1.

ICAM-2, like many other adhesion molecules, is known to play multiple roles in EC: leukocyte trafficking, inflammation and angiogenesis. These data now add a novel function, namely regulation of permeability. These findings may be particularly relevant to inflammation. In the ICAM-2 KO mice, thrombin-induced permeability was reduced compared to controls. Thrombin is known to have a significant remodelling effect over endothelial junctions, by acting through several pathways including small GTPases for review [36]. Our data suggests that ICAM-2 controls the cellular response to thrombin by regulating Rac1 activity. Future studies will investigate the role of ICAM-2 in permeability induced by other stimuli, and the possible cross-talk with other cell surface receptors in controlling barrier function.

## Conclusions

In conclusion, the data presented here describe a new role for the adhesion molecule ICAM-2 in regulating the localization of NCad in the early stages of monolayer formation and in the control of permeability. Based on these results, we propose a model where ICAM-2 supports early contact between EC by driving recruitment of NCad at the junctions. This model is similar to that proposed for nectins, cell adhesion molecules of the Ig superfamily which regulate the organization of adherens junctions by mediating early contact between epithelial cells and recruiting E-Cadherin to the junctions [50]. Because of the role of NCad in new vessel formation, these results suggest ICAM-2 might regulate permeability and angiogenesis at least in part through its ability to recruit NCad at the junctions.

## Methods

### Animals

All animal care and experimental procedures were performed under licence and complied with the UK Animals (Scientific Procedures) Act, 1986. ICAM-2 deficient mice [40] where backcrossed for 10 generations with C57/BL6 WT mice, supplied by Charles River Laboratories UK. WT and ICAM-2 deficient mice were 8-11 months old at the time of the experiments. Animals were maintained in standard condition at the Biological services Unit, Hammersmith Hospital, London, UK.

### Reagents and antibodies

Antibodies (Ab) against human ICAM-2, monoclonal Ab (mAb) BT-1 and polyclonal sc-1512, were obtained from Serotec and Santa-Cruz, respectively. mAb against mouse ICAM-2 (3C4) was obtained from Pharmingen. mAb against NCad (Cl 32) was purchased from BD Bioscience. mAbs against human VECad were purchased from BD bioscience (Cl75) and Pharmingen (Cl55-7H1). The mAb anti-mouse VECad (BV13) was kindly provided by Prof Elisabetta. Dejana (IFOM, Italy [51]). mAb PECAM-1 P2B1 was purchased from Santa-Cruz. mAb MJ7/18 anti-mouse endoglin was purchased from Chemicon. mAb Cl 23A8 anti-Rac1 was purchased from Transduction Laboratories. The anti-α-tubulin was obtained from SIGMA. The pBabe-Puro retroviral vector was a gift from Dr Aleksandar Ivetic (King’s College London, UK). PEGFP-C1 control, constitutive active V12 Rac1-GFP and dominant negative V12-N17 Rac1-GFP were kindly provided by Prof. Francisco Sanchez-Madrid (Universidad Autonoma de Madrid, Spain [33]. The PAK-GST construct was kindly provided by Prof. Anne Ridley (King’s College London, UK). Fluroscein isothiocyanate (FITC)-labelled albumin (Sigma, UK) solubilised in deionised water. Thrombin was purchased from Sigma-Aldrich (Poole, Dorset, UK) and solubilised in physiological saline.

### Generation and culture of mouse endothelioma lines

Primary murine cardiac endothelial cells (MCEC) were isolated from ICAM-2 deficient mice as previously described [52]. The released EC were isolated by positive endoglin selection using Mac Microbead system (Miltenyi Biotech). MCEC cells were cultured in DMEM supplemented with 10% fetal calf serum (FCS), 30ug/mL endothelial cell growth supplement (ECGS; BD Biosciences), 10 U/mL heparin, 2 mM L-glutamine, 100 IU/mL penicillin, and 0.1 mg/mL streptomycin). For all experimental assays, the cells were grown in DMEM media supplemented with 1% FCS.

Immortalization of primary MCEC cells was carried out with Polyoma middle T oncogene known to immortalize only the endothelial cells [24]. To generate cell lines expressing full length or mutant murine ICAM-2, the immortalized MCEC IC2 negative line was infected with the retroviral supernatant corresponding to the empty vector pBabe-Puro, pBabe-IC2, pBabe-IC2 ΔTAIL or pBabe-IC2 ΔERM at a multiplicity of infection of 0.025 to generate the following lines: IC2 neg, IC2 FL, IC2 ΔERM and IC2 ΔTAIL, respectively. Plasmids contruct methods were detailed in additional data section. Retroviral supernatants were generated by transfecting Phoenix ecotropic packaging cells (Orbigen Inc) with calcium phosphate according to the manufacturer’s protocol. 96 hours post-infection, cells were selected with puromycin (5 μg/mL, SIGMA). All the MCEC cells lines are polyclonal populations, thereby excluding a possible influence of vector integration on the phenotype.

### Transfection endothelioma cell lines

Endothelioma cell lines IC2 neg and IC2 FL were transfected with the plasmid pEGFP-C1 control, constitutive active Rac1 pEGFP-V12 or constitutive negative Rac1 pEGFP-V12-N17 with Lipofectamine2000 (Life technologies), according to the manufacturer’s procedure.

To generate cell line, KOIC2-PmT cells were infected with the retroviral supernatant pBabe-IC2 encoding full-length (FL) mouse ICAM-2 (IC2 FL), or empty vector pBabe (IC2 neg).

### Isolation and culture of HUVEC

HUVEC were isolated as previously described [53]. The use of human EC was approved by Hammersmith Hospital Research Ethics Committee (06/Q0406/21).

### Inhibition of expression by RNA interference

Human ICAM-2 siRNA (20 nM, Dharmacon) were delivered into 30% confluent HUVEC, cultured in EGM-2 media (Lonza-Cambrex) using AtuFECT01 lipid (1 μg/mL; Silence Therapeutics, Berlin, Germany) [54]. Non-targeting siRNA (Dharmacon) at the same concentration was used as a control. The following day, the transfection complex was replaced with medium M199 (containing 10% FCS and 15 μg/mL ECGS). After 6 hr, this was replaced with medium M199 (containing 10% FCS and 7.5 μg/mL ECGS) until analysis. Inhibition of NCad expression in IC2 FL was performed in medium DMEM 1% FCS using the same methods and siRNA concentration that was used for HUVEC. The siRNA sequences used were: NCad siRNA (UGUCAAUGGGGUUCUCCACdTdT) and GUGGAGAACCCCAUUGACAdTdT); scrambled siRNA (CAUGCGGAUUCGGAUUUUCdTdT and GAAAAUCCGAAUCCGCAUGdTdT).

### Permeability assay

Endothelial barrier assessment: Cells were grown on gold electrodes (in 8W1E arrays) and transendothelial electrical resistance (TEER) measured by real time impedance spectroscopy using ECIS (Applied Biophysics). Cells were grown to confluence in full medium and then switched into starvation medium containing 1% FCS for 48 h at which point TEER values were recorded. To assess responsiveness to vasoactive treatment, starved cells were stimulated with 1 U/ml thrombin (Sigma) and TEER changes measured after 10 min. To assess the role of Rac1, cells were infected with recombinant adenoviruses encoding constitutively active (V12) or dominant-negative (N17) myc-tagged Rac 1 [35] (a kind gift from Prof Anne Ridley, King’s College London, UK) at an m.o.i. of 400. After 4 h, virus was removed and the cells switched to starvation medium to assess barrier development.

In vivo permeability assay: Vascular permeability assay *in-vivo* was performed using the technique of measuring plasma protein extravasation in the mouse cremaster muscle [55]. Briefly, mice (WT and ICAM-2 deficient mice) were anaesthetised with ketamine (150 mg/kg; Ketaset, Fort Dodge Animal Health, Southampton, UK) and xylazine (7.5 mg/kg; Rompun, Bayer Healthcare, Newbury, UK). The jugular vein was exposed and cannulated with polyethylene tubing (PE10) for drug/dye administration.

The cremaster muscle was exteriorised [55,56] and gently laid across a Plexiglass viewing stage; and mounted on an Olympus “BW61WI” microscope with a water-immersion objective lens (magnification of 40×; LUMPlan, FI/IR, Olympus, Japan). Vascular permeability was measured by injecting FITC-albumin i.v. (0.25 mg per gram body weight) 10 min before recording. Five minutes prior to recording, thrombin (2 U/ml) was administered i.v. A snapshot of vessel fluorescence was taken using a block filter (excitation at 450—490 nm, emission at 535—620 nm) and a camera (model CoolSNAP HQ^2^, Photometrics, Tucson, AZ) coupled to a Windows XP-based computer for recording by Slidebook 4.2 (Intelligent Imaging Innovations, Inc., Denver, CO). Postcapillary venules with diameters of 20-40 μm were analyzed for a period of 10 minutes. Albumin leakage was quantified by measuring mean fluorescence intensity using ImageJ64 (National Institute of Health, USA). Average fluorescence intensity in three areas of equal size was measured: inside the vessel (Fl_in_), outside the vessel (Fl_out_) and background fluorescence in an area with no obvious leakage (bk). Albumin leakage was then determined as: [(Fl_out_ × bk)/(Flin × bk)] × 100%.

### Flow cytometry (FACS)

FACS analysis was performed using standard methods [52] and analyzed on an Epic XL-MCL flow (Beckman-Coulter).

### Cell proliferation assay

Cells were seeded onto 1% gelatin-coated 24 wells plates at 2500 cells/cm^2^, and counted using a hemocytometer in triplicate every day over 4 days.

### Western blot

Total protein was extracted using RIPA lysis buffer (10 mM sodium phosphate pH 8, 150 mM NaCl, 1% sodium deoxycholate, 1% NP40, 0.5% SDS, 1 mM PMSF, 10 mM NaF, 1 mM sodium orthovanadate) and protease inhibitor cocktail (SIGMA). Protein extracts were analysed by SDS-PAGE followed by immunoblotting using the indicated antibodies and detected using an enhanced ECL detection system (GE Healthcare). The intensity of the bands was quantified using alpha Innotech Chemi Imager software.

### Immunofluorescence microscopy

Cells were cultured on glass cover-slips, fixed with 4% paraformaldehyde, permeabilized with 0.4% Triton-X100 and immunostained with the indicated primary antibodies. Subsequent visualization was performed with AlexaFluor-conjugated Ab (Life technologies). For ICAM-2/VECad or VECad/NCad co-staining, VECad was labeled using the ZENON® kit (Life technologies), according to the manufacturer’s protocol. Nuclei were visualized using TOPRO-3 (Life technologies). Images were captured with a confocal microscope (LSM510 META; Carl Zeiss). Adobe Photoshop was used according to the guidelines to construct the confocal multi-channel images, to select specific regions of interest and to apply minor alterations to contrast and brightness uniformly across the entire figure panel.

### Rac pull-down assay

Rac1 activity was determined as previously described [17]. Briefly, cells were grown in reduced media for 48 hours and lysed in magnesium lysis buffer 1× (MLB) on ice. GTP loading of Rac was measured using 100 μg GST-PAK bound to glutathione beads (GE Healthcare), as previously described [57]. The detailed protocol of the Rac pull-down assay and preparation of lysis/wash buffers was provided by Upstate Biotechnology (Rac activity Assay kit).

### Statistics

Data are expressed as mean ± s.e.m. Comparisons between groups were performed with a two-tailed t-test using Prism software (GraphPad, San Diego, CA, USA).

## Abbreviations

EC: Endothelial cell; ERM: Ezrin-radixin-moesin; HUVEC: Human umbilical vein endothelial cell; ICAM-2 or IC2: Intercellular adhesion molecule-2; MCEC: Mouse cardiac endothelial cell; NCad: N-Cadherin; VECad: VE-Cadherin; AJ: Adherent junctions.

## Competing interests

The authors declare that they have no competing interests.

## Authors’ contributions

VA: performed research, generated reagents, analyzed data, contributed to the design of the research and to manuscript writing. NHD: performed research and contributed to data analysis and interpretation. RM and PT: performed in-vitro permeability assay and contributed to data analysis and interpretation. FG: performed in-vivo permeability assay and contributed to data analysis and interpretation. LOA: performed research and contributed to data analysis. GMB: contributed to data analysis and interpretation.DOH: contributed to data interpretation. JCM: contributed to experimental design and data interpretation. AMR: conceived the idea, designed the experimental plan, supervised research, carried out data analysis and interpretation, contributed to manuscript writing. All authors read and approved the final manuscript.

## Supplementary Material

Additional file 1: Figure S1 Analysis of N-Cad levels in confluent and sub-confluent HUVECs. N-Cad levels were measured by Western-Blot in HUVECs at 48h post-seeding, in sparse (5000 cells/cm2) and confluent condition (500000 cells/cm2). Quantification was performed by densitometry, normalized with GAPDH. Error bars indicate mean ± s.e.m., n = 3. t-test sparse vs confluent *p<0.05. Click here for file

Additional file 2: Figure S2 Analysis of ICAM-2 and N-Cad level after siRNA treatment. **A**- Analysis by Western-blot of ICAM-2 level after IC2 siRNA treatment from 24 to 72 h. Quantification of ICAM-2 Western-Blot was performed by densitometry, normalized with respect of α-tubulin. Error bars indicate mean ± s.e.m., n=5. t-test control vs IC2 siRNA *p<0.05, **p<0.01. **B**- Analysis by Western-blot of N-Cad level after N-Cad siRNA treatment from 24 to 96 h. Quantification of N-Cad Western-Blot was performed by densitometry, normalized with respect of α-tubulin. Error bars indicate mean ± s.e.m., n=5. t-test control vs N-Cad siRNA *p<0.05, **p<0.01,***p<0.001). Click here for file

Additional file 3: Figure S3 Distribution of VEC and PECAM-1 in HUVEC treated with ICAM-2 siRNA. VEC was visualized using mAb Cl55-7H1 followed by anti-mouse AlexaFluor 488 (Green) and PECAM-1 was visualised using mAb P2B1 anti-human PECAM-1 prelabelled with the Zenon^®^ mouse IgG1 555 kit (Red). Bar = 25 μm. Click here for file

Additional file 4: Figure S4 Endothelial characteristics of the endothelioma cell lines. **A**- Phase contrast image of WT Pmt, KOIC2 Pmt cell lines, showing that IC2 Pmt as well as have lost the typical cobblestone monolayer morphology and grow on top of each other whilst WT Pmt cell line have a cobblestone structure Bar = 150 μm. **B**- ICAM-2 over-expression restores tube formation on Matrigel. Cells were plated onto 48 wells (25000 cells/well) pre-coated with reduced growth factor Matrigel. Phase contrast pictures were taken 9 hours post-seeding using digital camera model DP50-CU (Olympus) connected to a Leitz labovert inverted microscope (Leica microsystems, objective x10). Bar=200 μm. **C**- Representative FACs profile of ICAM-2 and endoglin surface levels on IC2 neg, IC2 FL and HUVEC cells. Click here for file
