# Supplementary figures and images for: ICAM-2 regulates vascular permeability and N-cadherin localization through ezrin-radixin-moesin (ERM) proteins and Rac-1 signalling
Source: Cell Commun Signal. 2014 Mar 4;12:12. doi: 10.1186/1478-811X-12-12 (PMC4015342; doi:10.1186/1478-811X-12-12)

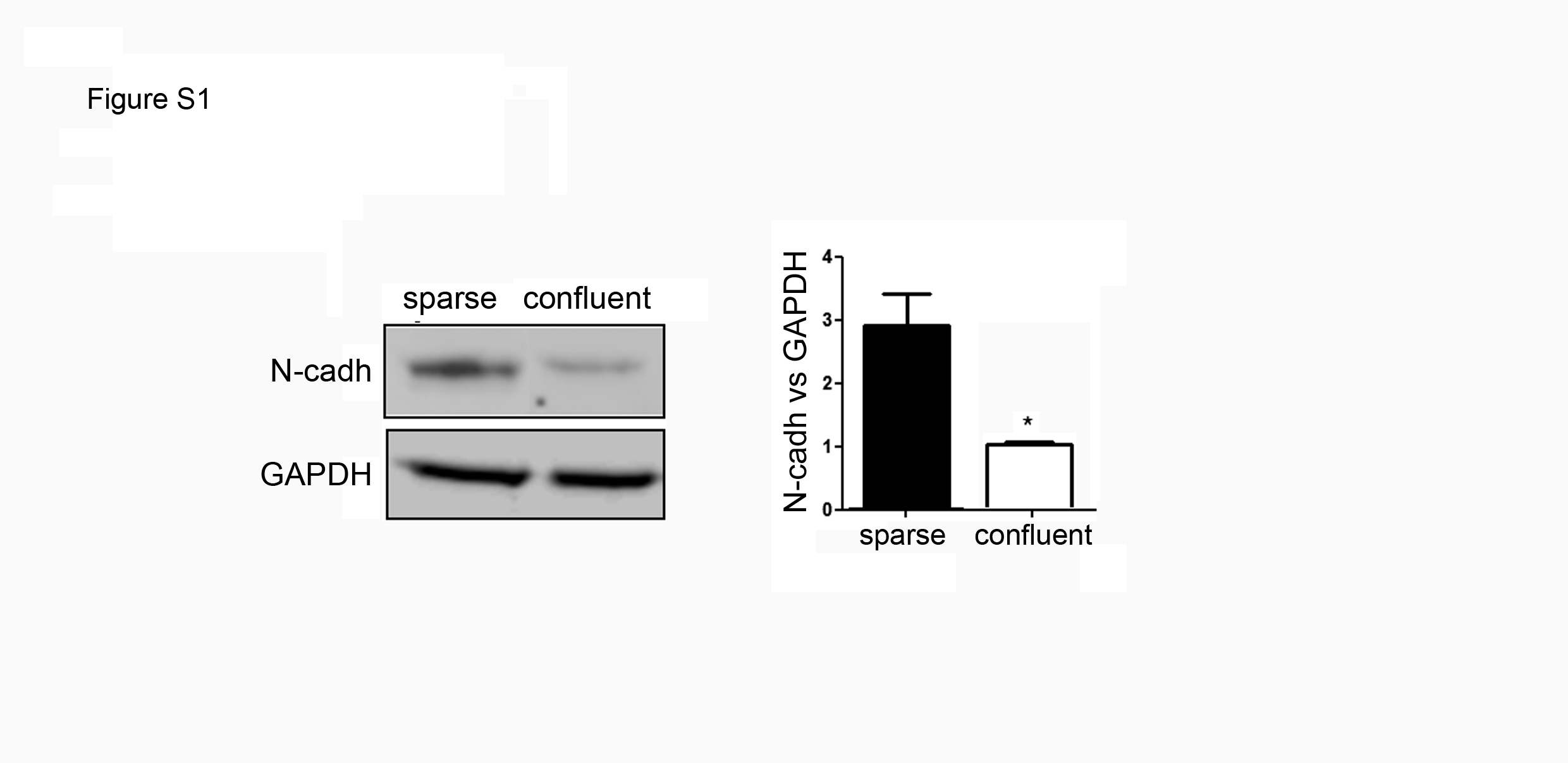

Supplement: Additional file 1: Figure S1 — Analysis of N-Cad levels in confluent and sub-confluent HUVECs. N-Cad levels were measured by Western-Blot in HUVECs at 48h post-seeding, in sparse (5000 cells/cm2) and confluent condition (500000 cells/cm2). Quantification was performed by densitometry, normalized with GAPDH. Error bars indicate mean ± s.e.m., n = 3. t-test sparse vs confluent *p<0.05. [file 1478-811X-12-12-S1.tiff]

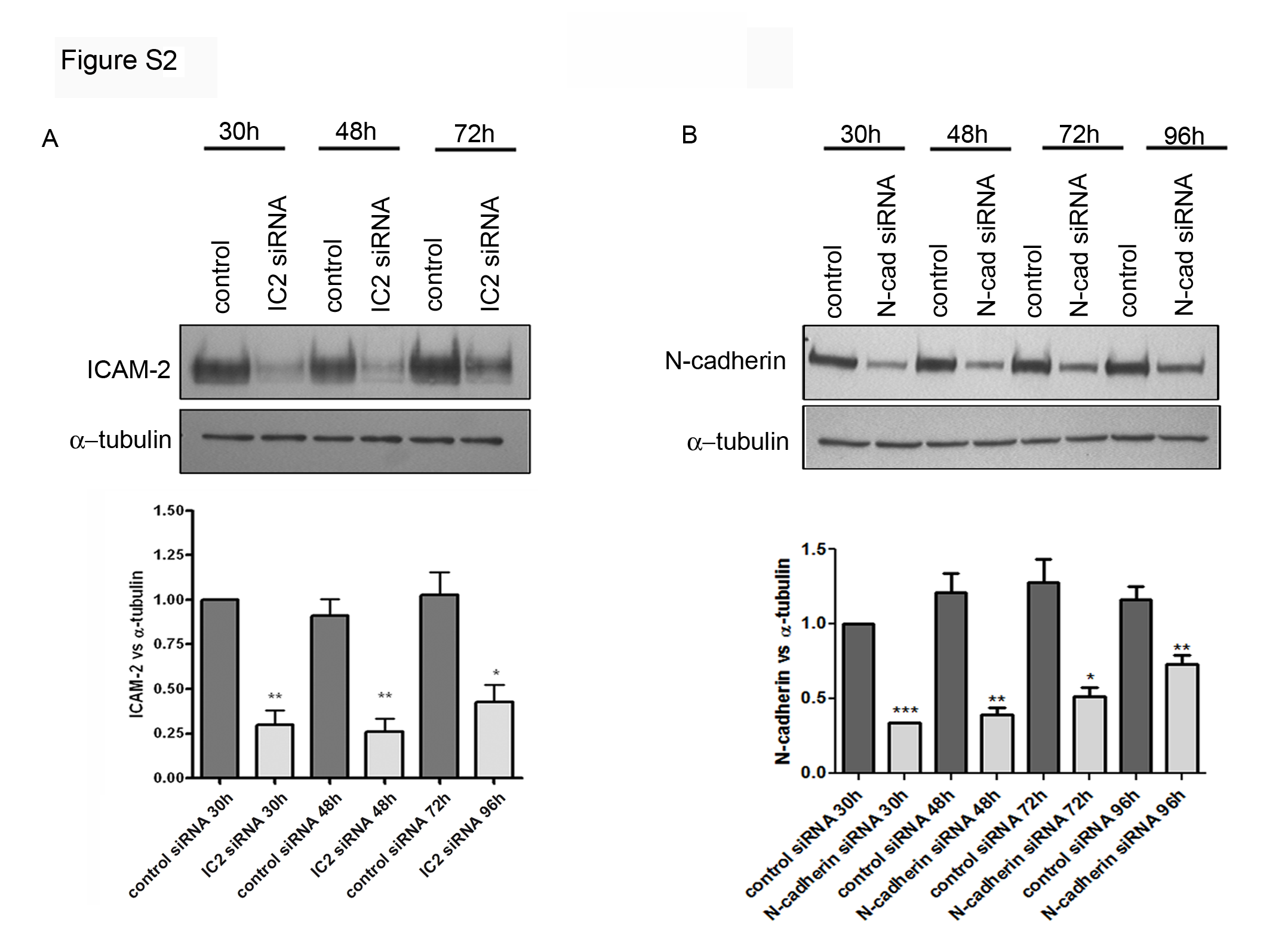

Supplement: Additional file 2: Figure S2 — Analysis of ICAM-2 and N-Cad level after siRNA treatment. A- Analysis by Western-blot of ICAM-2 level after IC2 siRNA treatment from 24 to 72 h. Quantification of ICAM-2 Western-Blot was performed by densitometry, normalized with respect of α-tubulin. Error bars indicate mean ± s.e.m., n=5. t-test control vs IC2 siRNA *p<0.05, **p<0.01. B- Analysis by Western-blot of N-Cad level after N-Cad siRNA treatment from 24 to 96 h. Quantification of N-Cad Western-Blot was performed by densitometry, normalized with respect of α-tubulin. Error bars indicate mean ± s.e.m., n=5. t-test control vs N-Cad siRNA *p<0.05, **p<0.01,***p<0.001). [file 1478-811X-12-12-S2.tiff]

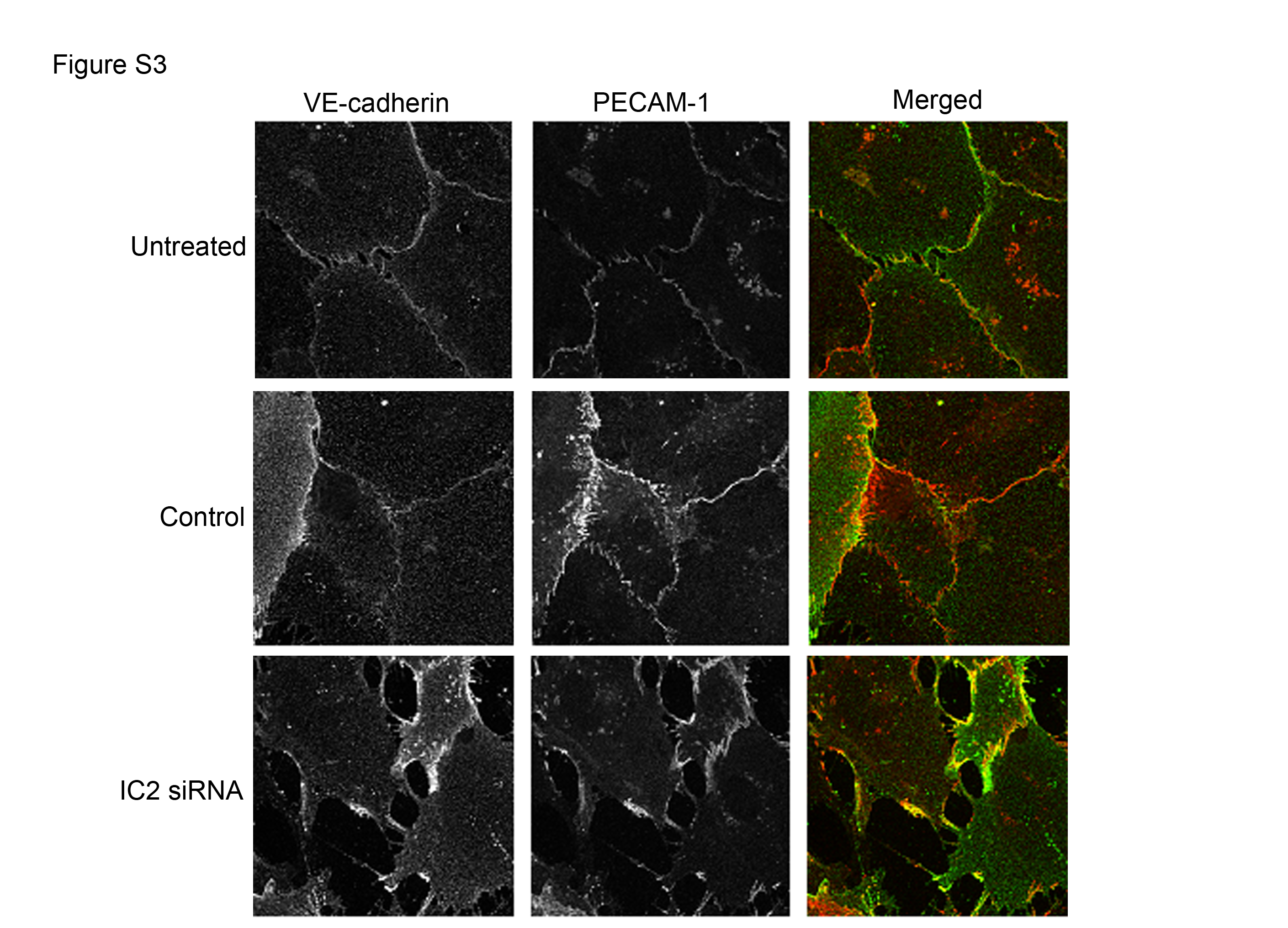

Supplement: Additional file 3: Figure S3 — Distribution of VEC and PECAM-1 in HUVEC treated with ICAM-2 siRNA. VEC was visualized using mAb Cl55-7H1 followed by anti-mouse AlexaFluor 488 (Green) and PECAM-1 was visualised using mAb P2B1 anti-human PECAM-1 prelabelled with the Zenon® mouse IgG1 555 kit (Red). Bar = 25 μm. [file 1478-811X-12-12-S3.tiff]

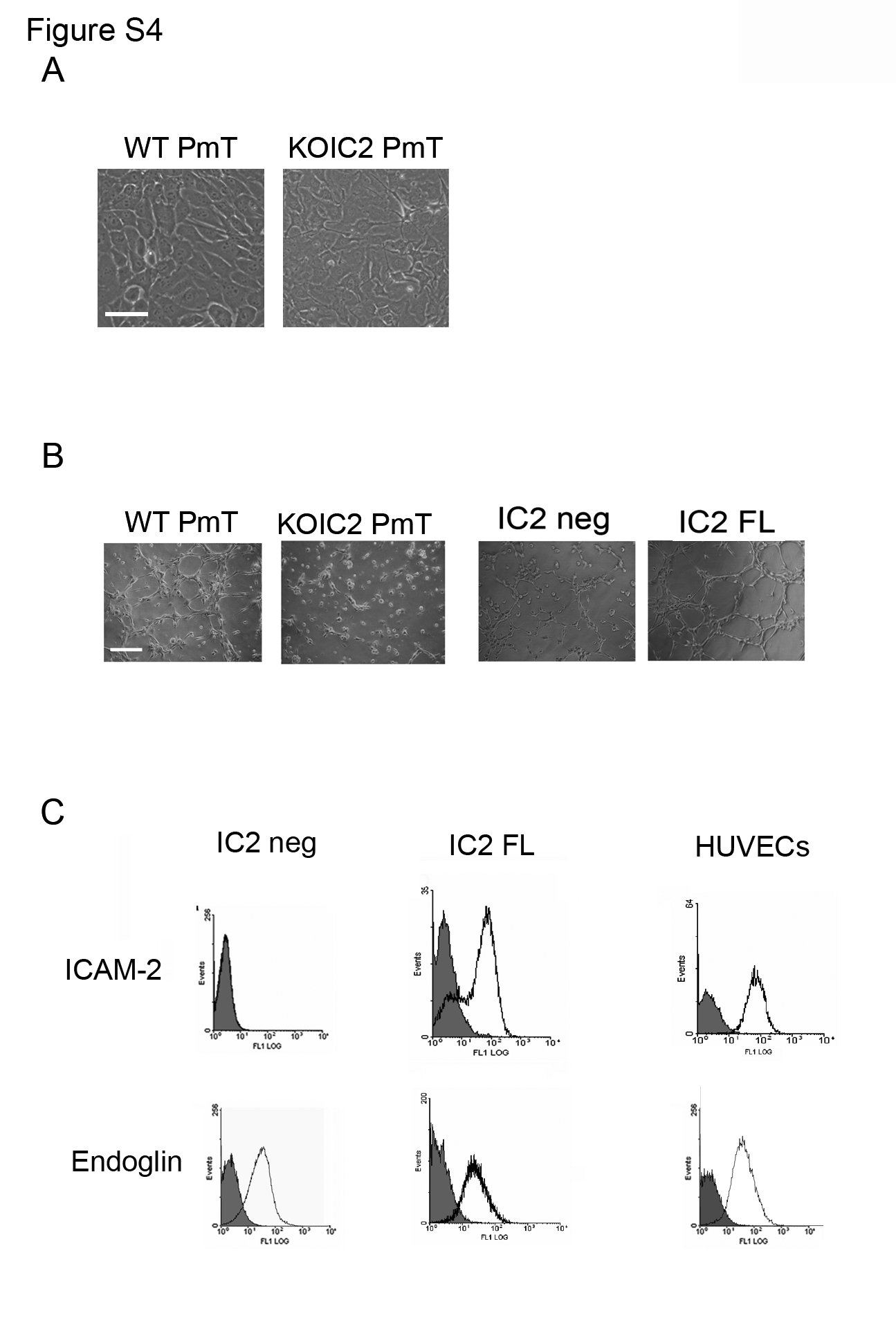

Supplement: Additional file 4: Figure S4 — Endothelial characteristics of the endothelioma cell lines. A- Phase contrast image of WT Pmt, KOIC2 Pmt cell lines, showing that IC2 Pmt as well as have lost the typical cobblestone monolayer morphology and grow on top of each other whilst WT Pmt cell line have a cobblestone structure Bar = 150 μm. B- ICAM-2 over-expression restores tube formation on Matrigel. Cells were plated onto 48 wells (25000 cells/well) pre-coated with reduced growth factor Matrigel. Phase contrast pictures were taken 9 hours post-seeding using digital camera model DP50-CU (Olympus) connected to a Leitz labovert inverted microscope (Leica microsystems, objective x10). Bar=200 μm. C- Representative FACs profile of ICAM-2 and endoglin surface levels on IC2 neg, IC2 FL and HUVEC cells. [file 1478-811X-12-12-S4.tiff]
